# Disjuncture between self-perceived and clinically assessed risk of HIV among gay, bisexual, and other men who have sex with men in Ontario and British Columbia, Canada

**DOI:** 10.1186/s12889-023-16065-0

**Published:** 2023-06-13

**Authors:** Oscar Javier Pico-Espinosa, Mark Hull, Mark Gaspar, Nathan Lachowsky, Daniel Grace, Robinson Truong, Saira Mohammed, Paul MacPherson, Kevin Woodward, Darrell H. S. Tan

**Affiliations:** 1 St Michael’s Hospital, Toronto, ON Canada; 2 BC Centre for Excellence in HIV/AIDS, Vancouver, BC Canada; 3 University of Toronto, Toronto, ON Canada; 4 University of Victoria, Victoria, BC Canada; 5 University of Ottawa, Ottawa, ON Canada; 6 McMaster University, Hamilton, ON Canada

**Keywords:** Pre-exposure prophylaxis, Gay, Bisexual, And other men who have sex with men, Preventive health services, Needs and demand

## Abstract

**Background:**

Self-perceived and clinically assessed HIV risk do not always align. We compared self-perceived and clinically assessed risk of HIV and the reasons for self-perceived low risk of HIV among gay, bisexual, and other men who have sex with men (GBM) from large urban centers in Ontario and British Columbia, Canada.

**Methods:**

Never PrEP users recruited from sexual health clinics or online, completed a cross-sectional survey between July/2019 and August/2020. We contrasted self-perceived HIV risk against criteria from the Canadian PrEP guidelines and participants were categorized as concordant or discordant. We used content analysis to categorize participants’ free-text explanations for perceived low HIV risk. These were compared with answers to quantitative responses about condomless sex acts and number of partners.

**Results:**

Of 315 GBM who self-perceived low risk of HIV, 146 (46%) were considered at high risk according to the guidelines. Participants with discordant assessment were younger, had less years of formal education, were more often in an open relationship and were more likely to self-identify as gay. Reasons for self-perceived low HIV risk in the discordant group were condom use (27%), being in a committed relationship/having one main partner (15%), having no or infrequent anal sex (12%) and having few partners (10%).

**Conclusions:**

There is a disjuncture between self-perceived and clinically assessed risk of HIV. Some GBM may underestimate their HIV risk and clinical criteria may overestimate risk. Bridging these gaps requires efforts to increase HIV risk awareness in the community, and refinement of clinical assessments based on individualized discussions between the provider and the user.

## Introduction

HIV pre-exposure prophylaxis (PrEP) is a highly effective intervention for HIV prevention [1–4] To access PrEP, potential users are commonly screened by healthcare staff based on eligibility criteria. In Canada, guidelines with such criteria were published in 2017 to help clinicians identify potential PrEP users.[3] According to these guidelines, PrEP is recommended to men who have sex with men (MSM) and transgender women (TGW) at high risk of HIV, namely, those who have had condomless anal sex in the past six months and any of the following: infectious syphilis or a rectal bacterial sexually transmitted infection (STI), particularly if diagnosed in the past 12 months; recurrent (at least twice) use of non-occupational post-exposure prophylaxis (PEP); a High-Incidence risk index (HIRI-MSM)[5] score equal to or higher than 11, and/or an ongoing sexual relationship with an HIV positive partner with substantial risk of transmissible HIV. However, persons who meet clinical criteria for PrEP might not perceive themselves at risk, resulting in either not seeking PrEP or declining it when offered.

The discrepancy between individual MSM meeting clinical criteria for PrEP and them not feeling at risk for HIV acquisition (which likely influences perceived need for PrEP) is an important barrier to PrEP scale-up [6–11]. Multiple studies comparing risk perception against clinical criteria have found a poor correlation between these two variables [12–15]. Possible reasons for this discrepancy may be differences in how GBM and clinicians assess risk: GBM often take into account cycles or fluctuations in their sexual behaviour [16] and consider various types of partnerships/relationship agreements to assess their risk of HIV [17]. In contrast, clinical criteria often use monogamy as a reference, ignore the nuances of different relationship agreements and tend to see sexual behaviour as a constant instead of as a dynamic aspect of life [18].

Underestimation of HIV risk can be explained by both population-level and individual-level factors. According to the risk homeostasis theory [19], people adjust their behaviour to changes in the environment. When the environment is perceived as safer, a decline in the perceived need for risk mitigation strategies may occur. For example, there is evidence of decreased HIV risk perception among MSM coinciding with wider PrEP availability,[20–22] which might be relevant in Canada, where PrEP uptake has been increasing [23, 24]. On the other hand, clinical screening criteria, which favor sensitivity over specificity in order to identify a larger pool of eligible individuals, have limitations [25–28]. For example, one criterion of the Canadian PrEP guidelines is a score of 11 on the HIRI-MSM, which has a sensitivity of around 80% and a specificity of around 45% for future HIV infection [5], meaning that a significant number of persons would be falsely considered at high risk of acquiring HIV using this cut-off.

Thus, such disjuncture between clinically assessed and self-perceived risk of HIV is expected and it is most likely the result of a combination of some individuals underestimating their risk and clinical criteria not being precise or nuanced enough. However, such disagreement becomes problematic when the two parts fail to reconcile. For instance, assuming that the clinically assessed risk for HIV is the standard and definite benchmark and that individuals underestimate their risk may be problematic because it risks perpetuating power imbalances and alienating people from accessing preventive services, particularly among populations that already have poor PrEP uptake as a result of systemic discrimination [29, 30].

Our objectives were to compare perceived and clinically assessed risk of HIV and to interpret the reasons given by never PrEP users for their self-perceived low risk of HIV in the context of their self-reported sexual behaviors.

## Methods

We used data from a cross-sectional community-based survey, part of the PrEP implementation project (PRIMP), a multicomponent study with the objective of scaling up PrEP uptake among gay, bisexual and other men who have sex with men (GBM) in urban Ontario and British Columbia, Canada. GBM were recruited through advertisements on dating apps, social media and sexual health clinics in five large urban centers in these two provinces (Toronto, Ottawa, Hamilton, Vancouver and Victoria). Additional inclusion criteria were being at least 19 years of age and being able to communicate in English. The age cut-off was chosen because the legal age for study participation in British Columbia is 19 years old or older. Workers and volunteers of community-based partner organizations were excluded. Recruitment took place between July 2019 and August 2020 and participants received a $10 CAD gift card. The target sample size for the survey was 250 participants in each city (1250 in total), based on its primary objectives of estimating PrEP awareness, acceptability and usage. However, the present study is a secondary analysis of the final dataset.

The survey included all the items needed to ascertain PrEP eligibility according to Canadian PrEP guidelines, with the exception of ‘ongoing sexual contact with a person living with HIV with detectable viral load’.[3] We included all the components of the HIRI-MSM questionnaire [5]: age, number of male partners, number of HIV positive partners, number of encounters involving condomless receptive anal sex, number of encounters involving condomless insertive anal sex with HIV-positive partners, use of methamphetamines and use of poppers, all in the past six months. The survey also asked about a history of infectious syphilis, rectal gonorrhea, rectal chlamydia and use of HIV PEP. Of note, although the guidelines recommend focusing on bacterial STIs acquired in the past year, we considered all previous STIs.

To define perceived risk of HIV, we asked, *“How would you rate your risk for HIV infection in the next year?”*, with response options “*low*”, “*high*” or “*unsure*”. Next, participants were asked why they chose that answer in a free-text field. In addition, participants were asked, *“Have you ever used PrEP?“* Only data from participants who had never used PrEP and who perceived their risk of HIV as low, were further analyzed in the present study.

Participants who self-perceived low risk yet were classified as being at high risk of HIV infection according to Canadian PrEP guidelines, were assigned to the “*discordant”* group. Participants who perceived their HIV risk as low and did not meet the criteria were assigned to the “*concordant”* group. We next compared the sociodemographic and sexual health characteristics of these two groups using Chi square tests or Fisher’s exact tests for categorical variables and Wilcoxon rank-sum tests for continuous non-normally distributed variables. Specific variables were: sociocultural background (obtained by combining the variables place of birth and ethnic identity), level of education (high school or less, college or technical education, bachelor’s degree and postgraduate education), relationship status (no regular partner, open relationship and closed relationship), how the participant primarily meets sexual partners (school/work/friends, websites/applications, bars/clubs and/or bath houses/sex parties/cruising areas), sexual orientation, extent of disclosure of their sexual orientation (not out at all, out to a few, out to half, out to most or out to all), proportion of gay, bisexual, and queer persons in their social network, frequency of HIV testing and access to a primary care provider.

Free text answers regarding reasons for feeling at low HIV risk were coded into categories using content analysis [31] by two authors independently (first and last authors) and disagreements in the codes were discussed to reach consensus. We included more than one code per individual whenever they provided more than one reason for low perceived HIV risk. We next calculated the proportions of responses in each of the categories for both the concordant and the discordant group. Finally, in post hoc exploratory analyses to further understand participants’ reasons for self-perceived low risk, we examined quantitative responses (condomless anal sex acts and number of sex partners) that could shed light on the qualitative explanations where possible, and compared these quantitative variables between the discordant and concordant groups. All analyses were completed using Stata 16.[32].

All participants gave their consent prior to initiating the survey. The study was approved by the Research Ethics Boards of St. Michael’s Hospital, Ottawa Hospital, St. Joseph’s Hospital, Toronto General Hospital, University of Toronto, University of British Columbia and University of Victoria.

## Results

In total, 1527 participants initiated the questionnaire and 567 were never PrEP users, of which 488 answered the questions about self-perceived risk and the Canadian PrEP guidelines criteria. Those with complete information (488 of the 567 never PrEP users), were more likely to have received less formal education (p < 0.01), be white (p < 0.01), have a primary care provider (p < 0.01), be gay (p < 0.01) and be out (p < 0.01) than the 79 individuals with incomplete data. Of the 488 never PrEP users with complete information, 315 (65%) perceived their risk for HIV infection as low, 65 (13%) as high, 69 (14%) were unsure and 39 (8%) preferred not to answer. Of the 315 participants who perceived themselves at low risk, 146 (46%) were categorized into the “*discordant*” group. The remaining 169 (54%) were categorized into the “*concordant*” group (Table 1).

**Table 1:** Proportion of participants with and without clinical indication for PrEP by category of self-perceived HIV risk

	PrEP not indicated†		PrEP indicated†	
Self-perceived HIV risk	N	%	n	%
Low	169	54%	146	46%
High	12	18%	53	82%
Unsure	34	49%	35	51%
Prefers not to answer	15	38%	24	62%
Total	230	47%	258	53%

†According to the Canadian PrEP guidelines [3].

We assessed how the 146 participants in the discordant group met the Canadian PrEP guidelines criteria. All reported condomless anal sex in the past six months, which is a required criterion according to the guidelines. For 96 participants (66%), PrEP eligibility was based only on a HIRI score ≥ 11; two (1%) had a history of syphilis only, and one (< 1%) had history of rectal gonorrhea only. Thirty-three (22%) had a HIRI-MSM score ≥ 11 plus another criterion, and 14 (10%) met three or four criteria. Since the HIRI-MSM score was a major reason for being defined as PrEP-eligible, we further explored which HIRI-MSM criteria were met among those with scores ≥ 11. Sixty-three out of 143 (44%) had receptive condomless anal sex (10 points according to the HIRI-MSM score [5]) and were between 18 and 48 years only (2–8 points), 22 (16%) had receptive condomless anal sex, were between 18 and 40 years and used poppers (3 points), 4 (3%) had receptive condomless anal sex and used poppers, and 54 (37%) had other combinations of criteria.

Discordant assessment of HIV risk was more common among participants who were younger (p = 0.001); had received less years of formal education (p = 0.031); were in an open relationship or did not have a regular partner (p = 0.005); self-identified as gay (p = 0.016); and met sex partners at bars/clubs (p = 0.010) (Table 2).

**Table 2:** Characteristics of the study participants stratified by being in the concordant (both perceived and clinically assessed low risk of HIV) or discordant group (perceived low risk of HIV and clinically assessed high risk of HIV).

	Concordant		Discordant		
**Age in years, median (Q1-Q3)**	35 (27–48)		31 (26–38)		z = 3.259 (p = 0.001*)
**Sociocultural background**	n	%	n	%	Chi2 = 4.410, Cramer’s V = 0.119, (p = 0.939)†
White born in Canada	86	51%	68	47%	
White born abroad	18	11%	16	11%	
Black born in Canada	4	2%	5	4%	
Black born abroad	3	2%	1	1%	
East Asian born in Canada	7	4%	5	4%	
East Asian born abroad	11	7%	11	8%	
Latin born in Canada	3	2%	2	1%	
Latin born abroad	8	5%	5	3%	
Indigenous people of Canada	2	1%	5	3%	
Other born in Canada	8	5%	9	6%	
Other born abroad	18	11%	19	13%	
Total	168		146		
**Education**					Chi2 = 8.873, Cramer’s V = 0.168, (p = 0.031)
High school or less	24	14%	11	8%	
College/technical	24	14%	38	26%	
Bachelor’s degree	74	44%	61	42%	
Postgraduate	45	27%	36	25%	
Total	167		146		
**Relationship status**					Chi2 = 12.949, Cramer’s V = 0.203, (p = 0.005)
No regular partner	101	60%	62	43%	
Open relationship	36	21%	56	39%	
Closed relationship	26	15%	23	16%	
Prefers not to answer	6	4%	3	2%	
Total	169		144		
**How they met sexual partners in the past 6 months**‡					
School, work, friends	37	22%	36	25%	Chi2 = 0.336, Cramer’s V = 0.033, (p = 0.562)
Websites, mobile applications	120	71%	113	77%	Chi2 = 1.662, Cramer’s V = 0.073, (p = 0.197)
Bars, clubs	29	17%	43	29%	Chi2 = 6.712, Cramer’s V = 0.146, (p = 0.01)
Bath house, sex parties, cruising	31	18%	34	23%	Chi2 = 1.169, Cramer’s V = 0.061, (p = 0.28)
No new sex partners in past 6 months	20	12%	12	8%	Chi2 = 1.122, Cramer’s V = 0.060, (p = 0.29)
Total*	169		146		
**Sexual orientation**					Chi2 = 12.917, Cramer’s V = 0.203, (p = 0.016)†
Gay	119	71%	124	85%	
Bisexual	38	23%	15	10%	
Pansexual	5	3%	4	3%	
Queer	2	1%	3	2%	
Other	4	2%	0	0%	
Total	168		146		
**Extent of disclosure of sexual orientation**					Chi2 = 6.607, Cramer’s V = 0.145, (p = 0.254)†
Not out at all	18	11%	7	5%	
Out to a few	31	18%	25	17%	
Out to half	15	9%	17	12%	
Out to most	27	16%	33	23%	
Out to all	75	44%	63	43%	
Prefers not to answer	3	2%	1	1%	
Total	169		146		
**Proportion of GBQ persons in social network**					Chi2 = 4.323, Cramer’s V = 0.119, (p = 0.229)
0–25%	81	51%	57	40%	
26–50%	33	21%	34	24%	
50–75%	30	19%	31	22%	
> 75%	16	10%	22	15%	
Total	160		144		
**Has a primary Care Provider**					Chi2 = 0.565, Cramer’s V = 0.042, (p = 0.745)†
Yes	120	71%	101	69%	
No	48	28%	43	29%	
Prefers not to answer	1	1%	2	1%	
Total	169		146		

*Wilcoxon rank-sum test. †Fisher’s exact test. ‡Multiple responses allowed

By definition, variables that were used in the classification of participants as discordant or concordant showed larger values in the discordant group: HIRI-MSM score (p < 0.001), total number of sexual partners (p < 0.001), number of HIV positive partners (p < 0.001), STIs (history of syphilis, p = 0.012; rectal chlamydia, p = 0.003; rectal gonorrhea, p = 0.002), use of methamphetamines (p < 0.001) and use of poppers (p < 0.001) (Table 3).

**Table 3:** Sexual health variables stratified by being in the concordant (both perceived and clinically assessed low risk of HIV) or discordant group (perceived low risk of HIV and clinically assessed high risk of HIV).

	Concordant		Discordant		p-value
**HIRI-MSM score, median (Q1-Q3)**	6 (3–8)		18 (15–21)		z = -15.023 (p < 0.001)
**Number sexual partners, median (Q1-Q3)**	2 (1–3)		3 (1–5)		z = -4.752 (p < 0.001)
**Number sexual partners HIV(+), median (Q1-Q3)**	0 (0–0)		0 (0–1)		z = -4.297 (p < 0.001)
**Syphilis diagnosis**	n	%	n	%	
Ever	15	12%	26	26%	Chi2 = 6.365, Cramer’s V = 0.164, (p = 0.012)
In the past year	4	3%	7	7%	Chi2 = 1.609, Cramer’s V = 0.083, (p = 0.205)
**Chlamydia diagnosis**					
Ever	32	23%	47	41%	Chi2 = 8.832, Cramer’s V = 0.187, (p = 0.003)
In the past year	13	10%	17	16%	Chi2 = 1.991, Cramer’s V = 0.090, (p = 0.158)
Rectal, ever†	3	9%	14	38%	Chi2 = 8.541, Cramer’s V = 0.344, (p = 0.003)
**Gonorrhea diagnosis**					
Ever	37	27%	54	44%	Chi2 = 8.906, Cramer’s V = 0.185, (p = 0.003)
In the past year	13	10%	21	19%	Chi2 = 4.455, Cramer’s V = 0.133, (p = 0.035)
Rectal, ever†	5	14%	22	48%	Chi2 = 10.062, Cramer’s V = 0.353, (p = 0.002)
**Use of PEP at least twice**	5	3%	9	6%	Chi2 = 1.896, Cramer’s V = 0.078, (p = 0.169)
**Methamphetamines use, past 6 months**	1	1%	13	9%	Chi2 = 12.846, Cramer’s V = 0.202 (p < 0.001)
**Poppers use, past 6 months**	29	17%	50	34%	Chi2 = 12.438, Cramer’s V = 0.199 (p < 0.001)
**Frequency of HIV testing**					
Once every 3 months or more often	25	16%	17	12%	Chi2 = 2.410, Cramer’s V = 0.089 (p = 0.300)
Once every 6 months or one year	97	60%	99	69%	
Once every 2 years or less often	39	24%	28	19%	

†Denominator is individuals ever having the infection. PEP: HIV post-exposure prophylaxis

Qualitatively analyzed groupings of reasons for low self-perceived risk of HIV are shown in Table 4. With the exception of condom use, there were no other statistically significant differences in the reasons for self-perceived low risk. Among respondents in the discordant group, the most common reason for self-perceived low risk of HIV was condom use (27%). This was followed by being in a committed relationship/having one main partnership (15%), serosorting and/or strategic positioning (14%), no or infrequent anal sex (12%) and having few sexual partners (10%). Importantly, there was variation within the categories generated. For example, in the ‘committed relationship/one main partner’ category, we included individuals who indicated some form of sentimental committed relationship or who expressed having a main partner despite indicating having sexual encounters with other types of partners.

**Table 4:** Reasons for perceived low risk of HIV stratified by being in the concordant (both perceived and clinically assessed low risk of HIV) or discordant group (perceived low risk of HIV and clinically assessed high risk of HIV).

Reasons for self-perceived low risk of HIV	Concordant		Discordant		p-value	Examples
	n	%	n	%		
Condom use	70	41%	39	27%	Chi2 = 7.488, Cramer’s V = 0.154, (p = 0.006)	“*I always use condoms*”, “*I generally use condoms*”
Committed relationship/one main partnership	18	11%	22	15%	Chi2 = 1.379, Cramer’s V = 0.066, (p = 0.240)	“*In a relationship*”, “*One partner. Only oral otherwise*”
No or infrequent (anal) sex	38	22%	17	12%	Chi2 = 6.388, Cramer’s V = 0.142, (p = 0.011)	*“Have minimal anal sex”, “Barely active”*
Serosorting / strategic positioning	25	15%	20	14%	Chi2 = 0.077, Cramer’s V = 0.016, (p = 0.782)	*“Very discriminating in partners”, “Discuss with person first”*
Few partners	22	13%	15	10%	Chi2 = 0.569, Cramer’s V = 0.043, (p = 0.451)	“*Same sexual partners as usual*”, “*Very few partners, only oral sex with strangers*”
Other†	5	3%	12	8%	Chi2 = 4.246, Cramer’s V = 0.116, (p = 0.039)	*“Unprotected sex with an individual who was taking PrEP.”, “Took PEP when exposed”*
Vague answer	6	4%	11	8%	Chi2 = 2.435, Cramer’s V = 0.088, (p = 0.119)	*“There are few risks”, “Very careful I guess”*
No response provided	25	15%	32	22%	Chi2 = 2.683, Cramer’s V = 0.092, (p = 0.101)	*-*
Total	169		146			

†Other include relying on partners being on PrEP, relying on partners reporting to be undetectable, using PEP if needed, and testing

To interpret the discordant respondents’ qualitative explanations for low risk in the context of responses from their peers in the concordant group, we compared quantitative responses about numbers of condomless anal sex acts and numbers of sexual partners between the groups, according to qualitative responses.

Of those who indicated using condom use as a reason for their low self-perceived risk of HIV: the median (Q1-Q3) condomless anal sex acts (CAS) was 2 (1–4) in the discordant group (n = 39), and 0 (0–0) in the concordant group (n = 70) (z = -9.84, p < 0.001). Of those who indicated having no or infrequent anal sex, the median CAS was 2 (1–5) in the discordant group (n = 17) and 0 (0–0) in the concordant group (n = 38) (z=-6.82, p < 0.001).

Of those who indicated having a regular partner as a reason for their low self-perceived risk of HIV: the median (Q1-Q3) number of partners was 1 (1–2) in the discordant group (n = 22), and 1 (1–1) in the concordant group (n = 18) (z=-1.25, p = 0.212). Of those who indicated having few partners, the median number of partners was 2 (1–3) in the discordant group (n = 13) and 2 (1–2) in the concordant group (n = 22) (z=-0.99, p = 0.324).

## Discussion

In this cross-sectional study, we found that 46% of never PrEP users who perceived themselves at low risk, were considered at high risk for HIV according to the Canadian PrEP guidelines. Our findings provide some insight on the characteristics of individuals whose self-perceived risk of HIV aligns with clinical criteria and of those for whom that is not the case. Reasons for self-perceived low risk included a combination of evidence-based actions and less reliable HIV mitigation strategies: condom use, avoiding anal sex, being in a committed relationship (monogamous or non-monogamous)/having one principal partner, limiting their number of sexual partners and being selective when choosing sexual partners. Respondents’ qualitative answers revealed nuances and contextual information not captured by the clinical criteria. However, we also observed some signs of HIV risk underestimation based on a wider distribution of objectively risky behaviors despite reporting similar risk mitigation strategies as the participants in the concordant group.

Our exploratory findings suggest that, compared to their peers, some respondents in the discordant group could be underestimating their HIV risk. The wider distribution of sex partners and condomless anal sex acts in the discordant group indicate that having a main partner, having few sex partners, using condoms, and not having anal sex frequently may mean different things to different people. These observations suggest that a subset of individuals who perceive themselves to be at low risk may do so on the basis of social/sexual norms that may differ substantially from both their peers and from clinical authorities. Of people in the discordant group, 27% reported condom use as a reason for self-perceived low risk, while reporting elsewhere in the questionnaire having had condomless anal sex in the past six months, perhaps explained by condom use with certain partners (e.g. casual) but not with others (e.g. regular partners). In addition, some GBM who had been diagnosed with syphilis, gonorrhea or chlamydia, self-perceived at low HIV risk, suggesting lack of awareness and an opportunity for greater health education. However, the assumption of lack of HIV risk awareness after STIs might be questionable since we did not collect nuanced information such as the contact being an individual with undetectable viral load or on PrEP, or did not consider the mode of transmission (e.g. syphilis acquired through oral contact).

Participants in the discordant group were more likely to be younger, self-identify as gay, be in an open relationship or not have a regular partner, and meet sexual partners in bars/clubs, suggesting that these might be groups to whom future efforts to enhance risk self-perception could be targeted. The younger age in the discordant group can be explained by the use of HIRI-MSM score as a criterion in the Canadian PrEP guidelines [3]. As pointed out in the results, 66% met clinical criteria based on their HIRI-MSM score; and among those, the largest proportion did so, based on the combination of young age (19–28 years of age) and at least one receptive condomless anal sex act, regardless of the context in which it occurred. For some perspective, HIV rates among GBM in Canada in the past 10 years have been very similar for the 20–29 and the 30–39 age groups [33], which does not correspond accurately to the scoring system that the HIRI-MSM score uses (8 points for the former and 5 points for the latter) [5]. The finding that more individuals in the concordant group self-identify as bisexual, correlates with evidence that bisexual men engage in anal sex less often than gay men; and among those who do, a significantly lower proportion engage in receptive anal sex. Therefore, it has been suggested that preventive efforts should not focus on this group [34]. We also found that open relationships were more common in the discordant group (p = 0.005), which aligns with observations that GBM in open relationships have agreements and allow for low risk sex outside of the relationship while reserving condomless anal sex for the main partnership, as described in more detail in the qualitative literature [17]. Considering the low risk of HIV transmission in those situations if such agreements are respected, [35] clinical criteria may overestimate the HIV risk by considering only partner number, rather than partnership-specific risk behaviors [18, 36]. However, a small proportion of new HIV infections among GBM do occur from the main sex partner [37, 38].

This complexity underscores how HIV risk can be both over- and under-estimated when considering quantitative sexual behavior data devoid of context. Therefore, it is important to recognize that clinical instruments, although helpful, have limitations and should be used as a starting point for a personalized assessment taking into consideration contextual factors including the ones discussed here. More stringent criteria, including a higher HIRI-MSM cut-off, would most likely increase the agreement between self-perceived and assessed risk of HIV. Although such a change would likely lead to higher acceptance of interventions like PrEP and to more efficient use of available resources [10, 11], it will exclude some individuals at risk, which is undesirable from a public health perspective.

## Limitations

First, our sample was primarily recruited at participating clinics, meaning that most were already engaged with healthcare services. Furthermore, participants with available data included people with more years of formal education, more likely to have a regular healthcare provider, more likely to be White, more likely to self-identify as gay, and more open about their sexual orientation, in contrast to those excluded due to incomplete data. We assume that if our sample were more diverse, including GBM with less years of formal education and without a regular healthcare provider, we would have perhaps observed a greater disjuncture between self-perceived and assessed risk of HIV infection. Second, history of STIs and PEP use was self-reported, which can lead to under-reporting [39]. Therefore, our findings may have under-estimated the discrepancy between self-perceived and assessed HIV risk. Third, we compared information derived from questions referring to the past six months, one year or further back in time, with self-perceived risk in the coming year, which may objectively differ considering the cyclical nature of sexual behavior. Fourth, we did not ask about sexual behavior separately for primary partners, regular non-primary partners, casual partners and anonymous partners. Having more detailed information about partner type would have provided a much better understanding of our findings. Last, there is risk for misclassification of self-perceived risk, since we only offered the options “high”, “low”, “unsure” and “prefer not to answer”. We believe this was a manageable way of asking but acknowledge its limited variability and that other ways of measuring it (e.g. with different parameters or other scales) could be more precise and/or could have led to different findings.

## Conclusions

In this sample of urban GBM in Canada, close to half of the participants who identified themselves at low risk of HIV were classified at high risk according to clinical criteria. In some cases, this disjuncture was due to risk overestimation by clinical screening criteria, but in other cases, due to risk underestimation because of GBM’s reliance on and confidence in behavioral HIV risk mitigation strategies. Asking GBM for more contextual information can explain and justify self-perceived low risk of HIV and offer opportunities for tailored risk reduction counseling. Clinical screening criteria have limitations and should not be the sole instrument to establish HIV risk, but instead, they should be used as a starting point for health education and clinical interventions for HIV prevention. Ultimately, a combined approach aimed at increasing awareness of the limitations of “objective” clinical parameters among providers, and at disseminating information among GBM about HIV risk mitigation strategies is necessary.

## Data Availability

The datasets generated and analyzed during the current study are not publicly available, since data analyses for other studies are ongoing. However, data are available from the corresponding author on reasonable request.
